# RevCAR-expressing immune effector cells for targeting of Fn14-positive glioblastoma

**DOI:** 10.1038/s41417-024-00766-8

**Published:** 2024-04-06

**Authors:** Haidy A. Saleh, Nicola Mitwasi, Liliana R. Loureiro, Alexandra Kegler, Karla Elizabeth González Soto, Lydia Hoffmann, Eugenia Crespo, Claudia Arndt, Ralf Bergmann, Michael Bachmann, Anja Feldmann

**Affiliations:** 1 Helmholtz-Zentrum Dresden-Rossendorf, Institute of Radiopharmaceutical Cancer Research, Department of Radioimmunology, Bautzner Landstraße 400, D-01328 Dresden, Germany; 2 Mildred Scheel Early Career Center, Faculty of Medicine Carl Gustav Carus, Technische Universität Dresden, D-01307 Dresden, Germany; 3 Department of Biophysics and Radiation Biology, Semmelweis University, Budapest, Hungary; 4 National Center for Tumor Diseases Dresden (NCT/UCC), Germany: German Cancer Research Center (DKFZ), Heidelberg, Germany; Faculty of Medicine and University Hospital Carl Gustav Carus, Technische Universität Dresden, Dresden, Germany; Helmholtz-Zentrum Dresden-Rossendorf (HZDR), Dresden, Germany; 5 German Cancer Research Center (DKFZ), Heidelberg, Germany; 6 German Cancer Consortium (DKTK), Partner Site Dresden, Dresden, Germany

**Keywords:** Drug development, Tumour immunology

## Abstract

In recent studies, we have established the unique adapter chimeric antigen receptor (CAR) platform RevCAR which uses, as an extracellular CAR domain, a peptide epitope instead of an antibody domain. RevCAR adapters (termed RevCAR target modules, RevTMs) are bispecific antibodies that enable the reversible ON/OFF switch of the RevCAR system, improving the safety compared to conventional CARs. Here, we describe for the first time its use for retargeting of both T and NK-92 cells. In addition, we describe the development and preclinical validation of a novel RevTM for targeting of the fibroblast growth factor-inducible 14 (Fn14) surface receptor which is overexpressed on Glioblastoma (GBM) cells, and therefore serves as a promising target for the treatment of GBM. The novel RevTM efficiently redirects RevCAR modified T and NK-92 cells and leads to the killing of GBM cells both in vitro and in vivo. Tumor cell killing is associated with increased IL-2, TNF-α and/or IFN-γ secretion. Hence, these findings give an insight into the complementary potential of both RevCAR T and NK-92 systems as a safe and specific immunotherapeutic approach against GBM.

## Introduction

Glioblastoma (GBM) is a highly aggressive malignant brain tumor with an incidence rate of 0.59 to 5 per 100,000 population [1]. The current standard therapy of GBM starts with surgery followed by chemo- and/or radiotherapy. Despite these aggressive treatments, the patient’s prognosis is poor [2, 3] underlining the urgent need for novel treatment options. Immune effector cells genetically modified to express chimeric antigen receptors (CAR) have shown remarkable success in the treatment of hematological malignancies which so far lead to the approval of six CAR products by the American Food and Drug Administration (FDA) [4]. This encourages an application of CAR-expressing immune effector cells also for the treatment of GBM.

Conventional CARs represent artificial receptors mainly containing three domains: (I) an extracellular anti-tumor-associated antigen (TAA)-binding domain [5, 6], (II) a transmembrane domain, and (III) an intracellular signaling domain [7, 8]. The extracellular antigen binding domain is most commonly constructed by fusion of the variable heavy (V_H_) and light (V_L_) chain domains of an antibody (Ab), which results in a single chain fragment variable (scFv) [6].

First generation CARs use an immunoreceptor tyrosine-based activation motif (ITAM) taken from the ζ-chain of the CD3 T cell receptor (TCR) complex for activation [9]. In order to improve the efficacy and survival of CAR T cells, second and third generation CARs were developed [9]. Besides the activation signaling domain, second generation CARs contain one additional co-stimulatory domain while third generation CARs contain even more co-stimulatory domains [9]. The costimulatory domains are taken from co-stimulatory receptors (e.g. CD28 or 4-1BB) [9].

The obvious purpose of adoptively transferred conventional CAR T cells is to recognize and eliminate all cells expressing the target recognized by the extracellular binding domain [10]. As TAAs are usually not exclusively expressed on tumor cells, CAR T cells will also attack healthy tissues known as on-target/off-tumor toxicity [11]. Moreover, the conventional CAR designs do not enable OFF and ON switching of the T cell activity in case severe side effects occur, which limits their application especially for the treatment of solid tumors [11]. In order to add a safety switch for the steering of CAR T cells, the adapter CAR technology was developed [11–14]. In contrast to conventional CARs, most adapter CARs have an extracellular Ab-derived epitope-binding domain which does not directly recognize a surface antigen on a tumor cell, but requires a linking molecule called target module (TM) for cross-linkage and activation of anti-tumor activity [11, 14–16]. Typically, a TM is a bifunctional fusion molecule consisting of the epitope recognized by the extracellular scFv of the adapter CAR fused to a binding moiety which is directed against the cell surface antigen of a target cell [14–16]. Consequently, adapter CAR immune cells are inactive in the absence of a TM and thus, their activity can be pharmacologically regulated [15]. One of these adapter CAR platforms is the UniCAR system which was developed by us [17, 18]. It shows high functionality and allows “safety switching” not only in pre-clinical studies but also in early clinical trials [12, 11, 19–21]. So far, we established two different UniCAR systems [17, 20, 22, 23]. These UniCAR immune cells recognize either the peptide epitope E5B9 or E7B6, respectively [14]. Both epitope sequences were taken from the primary amino acid sequence of the nuclear antigen La/SS-B [24, 25]. The epitopes are cryptic and not accessible in native La protein [24, 25]. Moreover, no autoimmune response against these epitopes were detected in autoimmune patients or in healthy individuals [14, 26]. Therefore, we believe that these epitope sequences have little if any immunogenicity [14, 26]. The extracellular binding domains of the two different UniCARs are derived from the respective anti-La monoclonal Abs (mAbs) La5B9 and La7B6 [14]. Unfortunately, the variable heavy and light chain portions of the extracellular CAR domain does not only fold in the expected way forming an intramolecular interaction but can also interact with the respective scFv chains of a neighboring CAR leading to a dimeric or even oligomeric CAR complex. These complexes can increase the tonic signaling of the CAR T cell [27].

In order to overcome the problem of tonic signaling, we have, more recently, presented a novel adapter CAR platform which we termed reverse CAR (RevCAR) system [15, 16, 28, 29]. In RevCARs, the extracellular Ab-derived binding domain of the respective UniCAR is replaced by the corresponding peptide epitope [15]. Vice versa, the peptide epitopes of the UniCAR TMs are replaced by the scFvs of the anti-La mAbs La5B9 or La7B6 [15]. Consequently, the RevTMs represent bispecific Abs (bsAbs) directed on the one hand to the peptide epitope (E5B9 or E7B6) and on the other hand to the cell surface antigen of the target cell [15]. Until now, we have shown proof-of-concept for functionality of both E5B9 and E7B6 RevCAR systems for targeting of prostate and colorectal cancer cells, as well as, acute myeloid leukemia cells [15, 16, 28]. A first clinical trial started recently targeting AML blasts with an anti-CD123 RevTM (NCT05949125). As their extracellular peptide domain cannot oligomerize, RevCARs cannot trigger tonic signaling comparable to scFv based extracellular domains [15].

In previous studies, we had shown that UniCARs cannot only be expressed in T but also in NK-92 cells [17, 19, 20, 22, 23]. An expression in NK or NK-92 cells is, however, not yet proven for RevCAR constructs. As CAR NK cells can be prepared from allogenic NK cells or the NK-92 cell line with low risk for graft versus host disease (GvHD), they could be used as “off-the-shelf” cell product [30, 31]. In addition, CAR NK cells have a short life span and, in comparison to T cells, different cytokine profile, accounting for a lower risk of on-target/off-tumor toxicity and cytokine release syndrome (CRS) [30, 31].

Up to now, different antigens, such as GD2, HER2, and EGFRvIII have been described as possible targets for GBM [32, 33]. In addition, the fibroblast growth factor-inducible 14 (Fn14) receptor represents a promising target for CAR therapy, since Fn14 itself and its cytokine ligand, the tumor necrosis factor-like weak inducer of apoptosis (TWEAK), have been detected as key players in proliferation, migration, invasion, and resistance to chemotherapy in various solid tumors, including GBM [34, 35]. Bearing in mind that Fn14 is also expressed on healthy tissues, although in lower levels than in solid tumors or injured tissues [36], the use of adapter CAR technologies should be superior over conventional CAR systems because the adapter CAR T cells can be switched off and thus diminishing on-target/off-tumor toxicity [11]. Therefore, we here describe for the first time the development of two novel RevTMs and their successful application for targeting of Fn14-positive GBM cells both in vitro and in vivo using switchable RevCAR T and RevCAR NK-92 cells.

## Materials and methods

### Cell lines

The NK-92 cell line was purchased from the DSMZ (Braunschweig, Germany) and cultured in X-VIVO medium (Lonza, Cologne, Germany) with 5% human plasma (German Red Cross, Dresden, Germany) and 400 IU/mL IL-2. The 3T3, HEK 293 T, Nalm6 and U343 were purchased from the American Type Culture Collection (ATCC, Manassas, VA, USA), while the U251 cell line was provided by Prof. Dr. Dieter Kabelitz, (Christian-Albrechts-Universität zu Kiel (CAU), Kiel, Germany). These cells were cultured in DMEM media with 10% fetal bovine serum (FBS) and incubated at 37 °C in and 5% CO_2_. For functional assay purposes, U343, U251, HEK293T, and Nalm6 cells were transduced by using lentivirus to express the firefly luciferase (Luc). All cells are regularly tested for mycoplasma.

### Design of the RevCAR construct

The second generation RevCAR construct is based on the ITAM of the intracellular ζ-chain of the CD3 complex (CD3z signaling motif) connected to the costimulatory domain, transmembrane region, and extracellular hinge domain of CD28 and a peptide epitope (E5B9 or E7B6). A human IL-2 derived signaling peptide (SP) is located N-terminally to allow the transport of the molecules to the cell surface. Following the RevCAR gene, a T2A protease cleavage site (Thosea asigna virus) and the EGFP expression marker are included [15]. Thus, transduction efficiency can be assessed based on the EGFP expression.

### Isolation of primary human T cells and transduction of RevCAR T cells

Primary human T cells were isolated from buffy coats of healthy donors which were obtained from German Red Cross after written consent from volunteers. The local ethics committee of the Medical Faculty Carl Gustav Carus, at the Technische Universität Dresden (Dresden, Germany) approved the research with human T cells (EK138042014). Using density centrifugation with Pancoll solution (1,077 g/ml) (PanBiotech, Aidenbach, Germany), primary T cells were isolated from Human Peripheral Blood Mononuclear Cells (PBMCs) using a pan T cell isolation kit according to the manufacturer’s instructions (Miltenyi Biotec, Bergisch Gladbach, Germany). Isolated T cells were stained with fluorescently labeled mAbs against human CD3 (#130-113-138), CD4 (#130-113-225), CD8 (#130-110-683) (Miltenyi Biotec). T cell lentiviral transduction procedure with RevCARs was done as described previously [15]. T cells were cultured with IL-15, IL-7 and IL-2 (Miltenyi Biotec) during transduction, but they were transferred to RPMI complete medium lacking these cytokines one day before any functional assay.

### Lentiviral transduction of RevCAR NK-92 cells

NK-92 cells were seeded at a density of 5×10^5^ cells/2 ml supplemented X-VIVO 10 media (Lonza Group, Basel, Switzerland). On the following day, the cells were mixed with lentiviral particles (MOI: 6) together with polybrene (16 μg/2 ml, Sigma-Aldrich Chemie GmbH, Taufkirchen, Germany) and the kinase inhibitor BX795 (8 μM, InvivoGen, Toulouse, France) for better lentiviral transduction efficiency of NK cells [37, 38]. Afterwards, cells were centrifuged in a 24-well plate for 60 min (1800 × g at 32 °C), followed by 6 h of incubation at 37 °C. The transduction mixture was then removed and replaced with fresh media. On the following day, NK-92 cells undergone a second transduction for 6 h at 37 °C. The second transduction mixture was replaced by fresh medium, and the cells were incubated at 37 °C. A third transduction was done on the following day. The efficiency of transduction was determined by EGFP expression marker using flow cytometry, where EGFP-positive cells were sorted by using a MACSQuant Tyto Cell Sorter (Miltenyi Biotec).

### Flow cytometry analysis and receptor or antigen density determination

The receptor density of RevCARs (expressed on transduced T or NK-92 cells) and the Fn14-antigen (on cancer cells) was determined using QIFIKIT® (Agilent, Santa Clara, CA, USA) as described by the manufacturer [15]. The expression of the E5B9 and E7B6 epitope on either T or NK-92 cells was determined by using the anti-La mAb La5B9 and La7B6, respectively, as described before [39]. In brief, Fn14 expression on cancer cells was determined by staining with an anti-human CD266 mAb (#314102; clone ITEM-4, BioLegend, San Diego, CA, USA), followed by a goat-anti-mouse IgG-AlexaFlour 647™ (#A21235; Thermo Fisher Scientific, Dreieich, Germany). As a control, mouse IgG2b isotype (#400302; clone MPC-11; BioLegend, San Diego, CA, USA) was used. For detection of Fn14-specific RevTM binding, 1×10^5^ target cells were incubated with RevTMs (40ug/ml) for 1 h at 4 °C, followed by incubation with an APC-conjugated anti-His mAb (#130-119-820; clone GG11-8F3.5.1, Miltenyi Biotec). All samples were analyzed using a MACSQuant® Analyzer and MACSQuantify® software (Miltenyi Biotec).

### Expression and purification of recombinant RevTMs

The general structure of RevTM has been previously described [15]. The sequence of anti-Fn14 V_L_ and V_H_ chain domains were based on a previously published Fn14 mAb P4A8 (patent EP2294089A2) [40]. The complete sequences of the Fn14-specific RevTMs (Fn14-5B9 or Fn14-7B6) were designed using SnapGene, and ordered and synthesized by Eurofins Genomics GmbH, Ebersberg, Germany. The DNA was then cut out with *Nhe*I/*Mes*I restriction enzymes and each inserted separately into the *Xb*aI/*Hpa*I-digested lentiviral vector p6NST50. 3T3 cell lines were transduced with lentiviral particles encoding the respective RevTM to induce stable expression. Afterwards, the RevTMs were purified from cell culture supernatants via Strep-Tactin®XT 4Flow® column according to manufacturer’s instructions (Fischer scientific, Schwerte, Germany). For the determination of the yield and purity, purified RevTMs were separated via SDS-PAGE and analyzed using Coomassie Brilliant Blue™ staining (Serva, Heidelberg, Germany) or immunoblotting as described before [15, 41].

### Cytotoxicity assay

In order to determine the RevCAR T and NK-92 cell-mediated tumor killing, a luminescence-based assay was performed as previously described [41]. For that purpose, U251 and U343 cell lines expressing firefly luciferase (U251 Luc and U343 Luc) were used [29]. 2×10^4^ RevCAR T cells were co-cultured with 4×10^4^ tumor cells (E:T ratio of 1:2) in the absence or presence of 50 nM or of a concentration gradient range of the respective RevTM for 20 h as indicated in each experiment. On the other hand, NK-92 cells were co-cultured with tumor cells at E:T ratios of 10:1, 5:1, and 2:1. Thus, 5×10^4^, 2,5×10^4^ or 1×10^4^ RevCAR NK-92 cells were incubated, respectively, with 5×10^3^ tumor cells in the absence or presence of 50 nM or of a concentration gradient range of the respective RevTM for 4 h as indicated in each experiment. After the indicated time of incubation, the luminescence was measured and the specific lysis was calculated as described previously [41].

### Cytokine measurement

For cytokine determination, tumor cells were co-cultured with RevCAR T cells for 18-20 h or RevCAR NK-92 cells for 4 h in the absence or presence of the corresponding RevTM (50 nM). Then, cell culture supernatants were collected and quantified using an ELISA kit (BD Biosciences, Heidelberg, Germany) according to the manufacturer’s instructions.

### In vivo experiment

In vivo co-injection experiment was performed in three groups of nine weeks-old female NXG-immunodeficient mice (NOD-Prkdcscid-IL2rgTm1/Rj, JANVIER LABS, Le Genest-Saint, France) with five mice in each group allocated by simple randomization without blinding. The first group was injected with 10^6^ U251 Luc cells, the second group with a mixture of 10^6^ U251 Luc cells with 10^6^ RevCAR NK-92 cells at a 1:1 ratio, and the third group with the same mixture as the second group but with the addition of 150 pmol of RevTM Fn14-5B9 in a total volume of 100 µl in PBS. Respective cell suspensions were administrated subcutaneously in the right flank of the mice. For optical imaging, mice were anesthetized as previously described [16, 41], and injected with 200 µl XenoLight D-Luciferin Potassium salt (15 mg/ml) (PerkinElmer LAS GmbH, Rodgau, Germany) for 10 min. With exposure time of 2 min, luminescence was detected using the In Vivo Xtreme Imaging System (Bruker, Bremen, Germany) over a period of four days. Animal experiment was conducted in accordance to the guidelines of the German Regulations for Animal Welfare, and approved by the local Ethical Committee for Animal Experiments (reference number DD24.1-5131/449/67).

### Statistical analysis

Statistical analysis was accomplished using GraphPad Prism 9.0 (La Jolla, CA, USA). Statistical significance was determined as mentioned in figure legends. P-values below 0.05 were considered statistically significant (*p* ≤ 0.05 (*), *p* ≤ 0.01 (**), *p* ≤ 0.001 (***), *p* ≤ 0.0001 (****)). Data are shown as mean values ± SD.

## Results

### Design and development of a RevCAR platform for targeting Fn14-expressing GBM cells

**Fig. 1:**
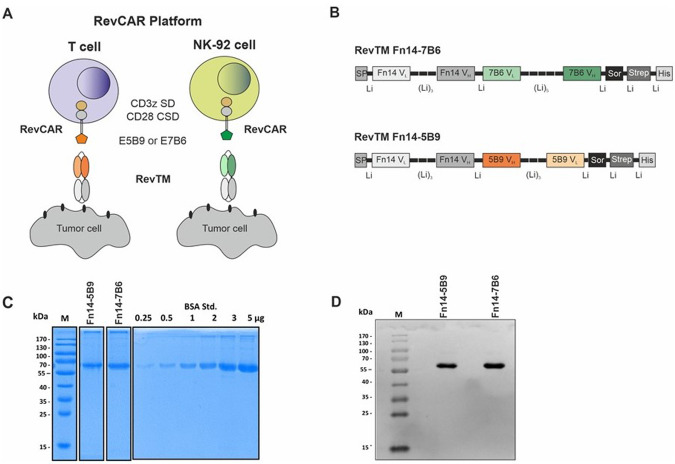
Schematic overview of RevCAR T and NK-92 cell systems. **A** Either RevCAR T cells or RevCAR NK-92 cells express reverse chimeric antigen receptors (RevCARs) that consist of an intracellular CD3z signaling domain (SD) as well as a CD28 co-stimulatory domain (CSD), in addition to a CD28 transmembrane and hinge region linked to an extracellular peptide epitope (either E5B9 or E7B6). RevCAR immune cells can be activated by RevTMs. **B** The novel designed RevTMs are bsAbs derived from the variable light (V_L_) and heavy (V_H_) chain domains of mAbs directed against either the TAA Fn14 or the RevCAR epitope E5B9 or E7B6. All domains are connected via peptide linkers (Li). Both RevTMs contain an N-terminal signal peptide (SP) and C-terminal Histidines (His) as well as a Strep-tag for their purification. After purification via their Strep tag, the RevTMs were separated by SDS-PAGE followed by (**C**) Coomassie Blue staining, or (**D**) immunoblotting on nitrocellulose membrane and detection via an anti-His mAb and an alkaline phophatase-conjugated anti-mouse Ab. Sor; sortase recognition site, M; molecular weight marker, BSA Std.; bovine serum albumin standard.

As shown in Fig. 1, the RevCAR platform consists of two components: On the one hand, the respective immune effector cell (T or NK-92 cells) genetically modified to express the artificial RevCAR, and on the other hand, a bispecific molecule called RevTM. The RevCAR is derived from a second-generation CAR construct and consists of an extracellular peptide epitope (E5B9 or E7B6), a CD28-derived hinge and transmembrane domain and an intracellular part containing the CD3z signaling domain (SD) and the CD28 costimulatory domain (CSD). In contrast to conventional CARs, RevCARs lack an antigen-binding moiety, and thereby use the RevTMs to recognize a TAA of interest.

To redirect either RevCAR-E5B9 or RevCAR-E7B6 T or the respective RevCAR NK-92 cells against Fn14-expressing GBM tumor cells, we developed two Fn14-specific RevTMs, namely Fn14-5B9 and Fn14-7B6 (Fig. 1A, B). Each of the RevTMs consists of two scFvs which on the one hand recognize one of the RevCAR epitopes (either E5B9 or E7B6), and on the other hand the Fn14 antigen. Expression cell lines were established and the respective RevTMs were purified (see Materials and Methods section). The purified RevTMs were analyzed by SDS-PAGE followed by staining with Coomassie Brilliant Blue™ or transferred onto nitrocellulose membranes and detected with an anti-His mAb. Both RevTMs were isolated in high yield and purity sufficient for further functional assays (Fig. 1C, D).

### Binding of Fn14-specific RevTMs to RevCARs and GBM target cells

**Fig. 2:**
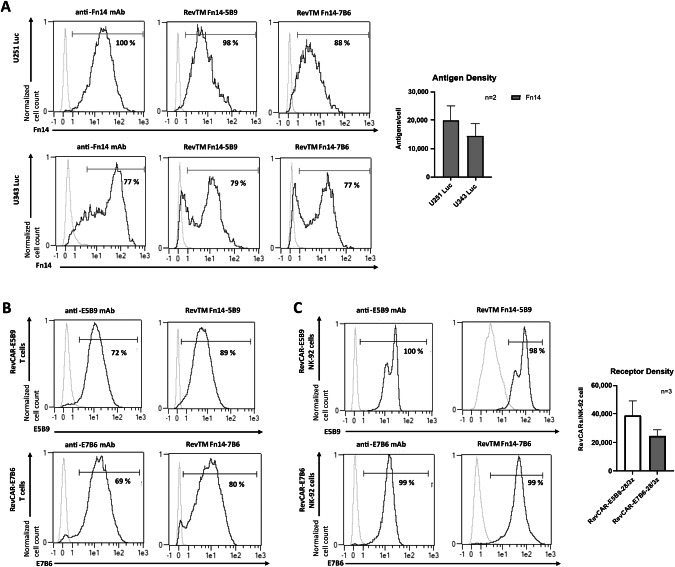
Binding of Fn14-specific RevTMs to GBM cells, RevCAR T cells and RevCAR NK-92 cells. **A** Fn14 expression on luciferase (Luc)-expressing U251 Luc and U343 Luc GBM cells was determined by staining with anti-Fn14 mAb (primary Ab) and AlexaFluor647-conjugated anti-mouse IgG (secondary Ab). The number of Fn14 antigens expressed per cell was detected by a bead-based flow cytometry assay (QIFIKIT). The binding of Fn14-specific RevTMs to Fn14 on both U251 Luc and U343 Luc cell lines was tested using APC-conjugated anti-His mAb. The expression of RevCAR receptors (E5B9 or E7B6) on transduced (**B**) T cells or (**C**) NK-92 cells was confirmed by the anti-La mAb La5B9 or La7B6, respectively, and the secondary Ab AlexaFluor647-conjugated anti-mouse IgG. In addition, the number of RevCAR receptors per NK-92 cell was determined. The binding of the Fn14-specific RevTMs to the respective RevCAR T or NK-92 cells was detected using an APC-conjugated anti-His mAb. **A**, **C** Quantitative data from three different experiments are shown as mean ± SD. **A**–**C** Flow cytometry data are displayed in histograms (light lines: negative control, dark lines: stained cells).

One first evidence for functionality of the newly developed bispecific RevTMs is their binding capability to the respective RevCAR epitope (E5B9 or E7B6) and to the Fn14-antigen. To evaluate the binding, we used the GBM cell lines U251 and U343 which were genetically modified to express the firefly Luciferase gene. Both cell lines naturally express Fn14, as shown by staining with an anti-Fn14 mAb. The number of Fn14 antigens per cell was estimated at around 20,000 for U251 Luc and 15,000 for U343 Luc cells (Fig. 2A). The two novel Fn14-specific RevTMs showed high binding to both GBM cell lines (Fig. 2A). Additionally, we assessed the expression of E5B9 or E7B6 on RevCAR T or RevCAR NK-92 cells using the anti-La mAb La5B9 or La7B6, respectively (Fig. 2B, C). Fn14-specific RevTMs were able to efficiently bind to their corresponding RevCAR epitopes on RevCAR T cells (Fig. 2B) or RevCAR NK-92 cells (Fig. 2C), with the latter expressing around 39,000 E5B9 or 25,000 E7B6 receptors/cell (Fig. 2C). For RevCAR T cells, the receptor density was already assessed in previous studies and ranged between 7000 and 15,000 RevCAR-E5B9 receptors/cell and 9,000 RevCAR-E7B6 receptors/cell [16, 29]. Overall, these findings confirm the binding of the novel RevTMs to both RevCAR effector cells and Fn14-expressing tumor cells.

### Targeting GBM cells with RevCAR T cells redirected by Fn14-specific RevTMs

**Fig. 3:**
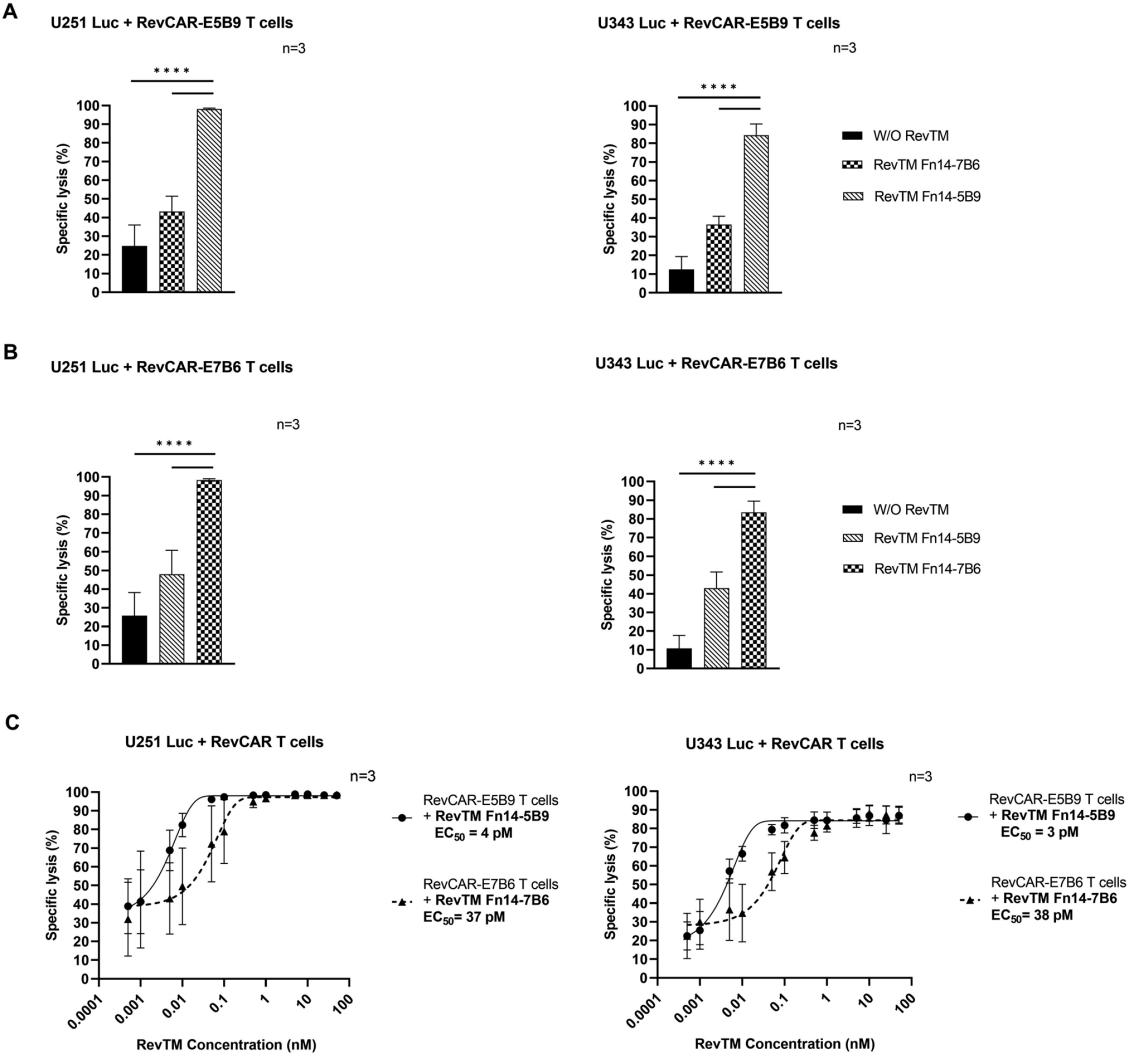
Specific lysis of GBM cells by Fn14-redirected RevCAR T cells. U251 Luc and U343 Luc cells expressing luciferase (Luc) were co-cultured with either (**A**) RevCAR-E5B9 or (**B**) RevCAR-E7B6 T cells at an effector to target (E:T) cell ratio of 1:2 in the absence or presence of RevTMs (50 nM) for 20 h. Cytotoxic activity was measured using a luminescence-based cytotoxicity assay. **C** Half-maximal effective concentration (EC_50_) of Fn14-specific RevTMs was estimated in a co-culture of tumor cells and RevCAR T cells with an E:T ratio of 1:2 (20 h of incubation). **A**–**C** Data are shown for three independent T cell donors and represented as mean ± SD (one-way ANOVA with Tukey multiple comparisons test, ****p < 0.0001; comparison to samples without (W/O) RevTM or with irrelevant RevTM).

To test the cytotoxic activity of RevCAR T cells redirected by the novel Fn14-specific RevTMs, we evaluated the specific lysis of the GBM cells by using a luminescence-based cytotoxicity assay. RevCAR T cells were co-cultured with U251 Luc or U343 Luc cells in the presence or absence of the respective Fn14-specific RevTM. The irrelevant RevTM containing the non-matching anti-RevCAR epitope binding domain was included as a negative control to verify the dependency of the RevCAR system on the cross-linkage of effector and tumor cells via a matching RevTM. As shown in Fig. 3, RevCAR-E5B9 (Fig. 3A) or RevCAR-E7B6 (Fig. 3B) T cells significantly lysed both GBM cell lines in the presence of their corresponding RevTM in comparison to the control conditions.

According to this data, the killing of GBM cells by RevCAR T cells is dependent on the presence of the respective RevTM. In order to assess the half-maximal effective concentration (EC_50_), we tested a concentration range of the RevTMs. As shown in Fig. 3C, we estimated an EC_50_ value in the pM range for both RevTMs. Confirmed with both U251 Luc and U343 Luc cells, RevTM Fn14-5B9 has 9- and 12.5-fold lower EC_50_ than RevTM-7B6, respectively.

Furthermore, to confirm the target dependency and specificity, we have tested the Fn14-directed RevCAR T cell system against HEK 293 T Luc cells that express lower levels of Fn14 or Nalm6 Luc cells lacking, almost completely, the Fn14 expression (Supplementary Fig. 1). As shown in Supplementary Fig. 1, in contrast to U251 Luc cells expressing a pronounced Fn14 level (approximately 20,000 antigens/cell) that can be highly effectively killed by the Fn14-directed RevCAR system, the Fn14 low-expressing HEK 293 T Luc cells or Fn14-negative Nalm6 Luc cells can hardly or not be killed at all using the same RevCAR-E5B9 T cells in the presence of the RevTM Fn14-5B9.

**Fig. 4:**
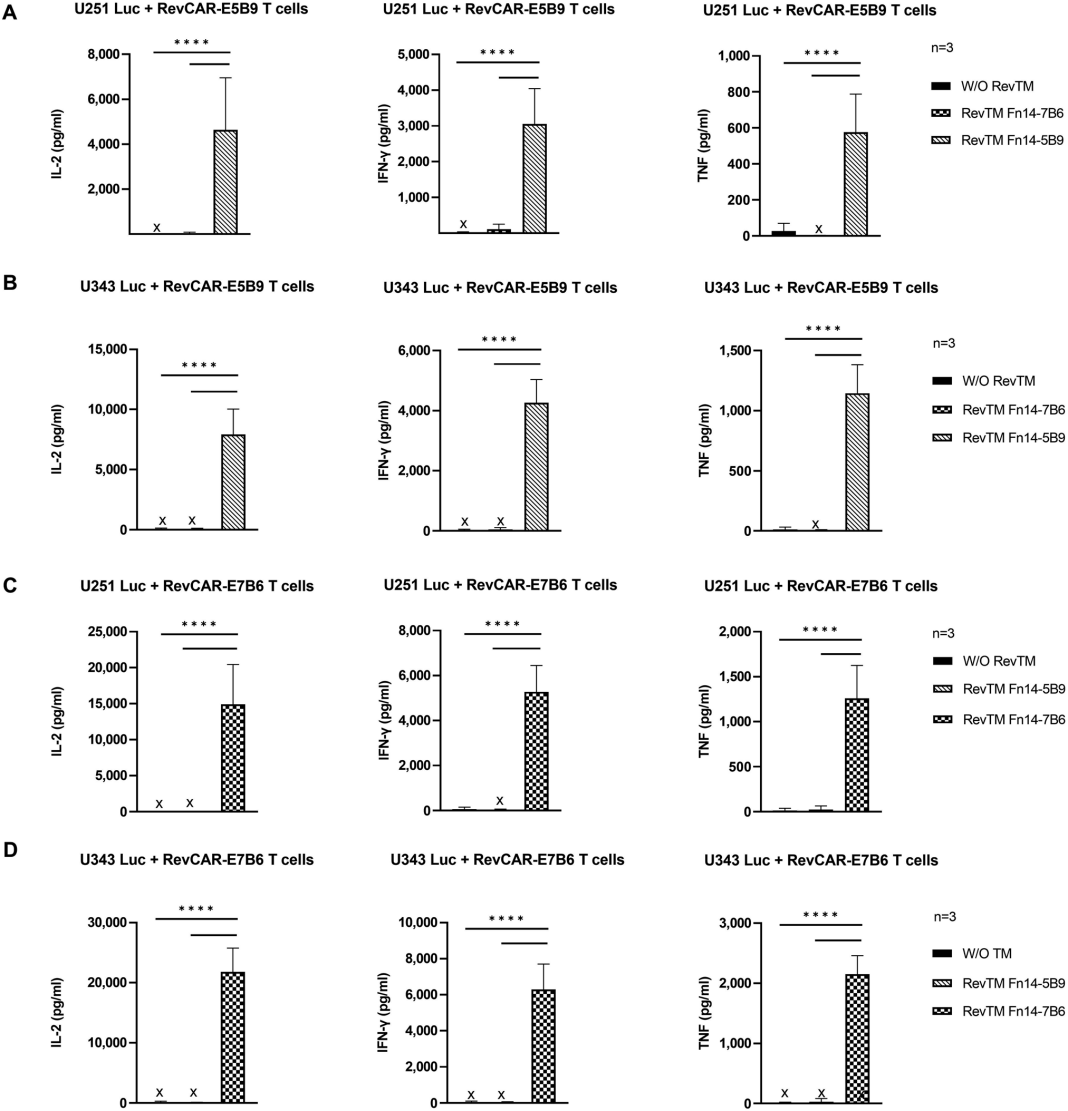
Cytokine secretion of Fn14-redirected RevCAR T cells. The pro-inflammatory cytokines IL-2, IFN-γ and TNF-α were detected using ELISA in supernatants from co-culture experiments of either (**A**) and **(C**) U251 Luc or (**B**) and (**D**) U343 Luc cells with either (**A**) and (**B**) RevCAR-E5B9 or (**C**) and (**D**) RevCAR-E7B6 T cells incubated at an E:T ratio of 1:2 in the absence or presence of matching RevTM or irrelevant RevTM (negative control) for 20 h. Results are shown as mean ± SD for three independent T cell donors (One-way ANOVA with Tukey multiple comparison test, ****p < 0.0001; comparison to samples without RevTM or with irrelevant RevTM). X; not detectable.

In addition to tumor cell killing, we measured the secretion of the pro-inflammatory cytokines IFN-γ, TNF-α and IL-2, which might be relevant on the one hand for potential side effects but also on the other hand for the anti-tumor response and the modulation of the TME. As shown in Fig. 4, only RevCAR T cells cross-linked with tumor cells via the matching Fn14-specific RevTM secrete significant amounts of cytokines in comparison to the controls, where no secretion of cytokines was observed in the control conditions. Interestingly, the RevCAR-E7B6 (Fig. 4C, D) induced higher cytokine release in comparison to the RevCAR-E5B9 (Fig. 4A, B) in both GBM cell lines.

In conclusion, the novel Fn14-directed RevCAR T cell system mediates an effective killing of GBM tumor cells and cytokine release in a target-specific and RevTM-dependent manner.

### Targeting GBM cells with RevCAR NK-92 cells redirected by Fn14-specific RevTMs

**Fig. 5:**
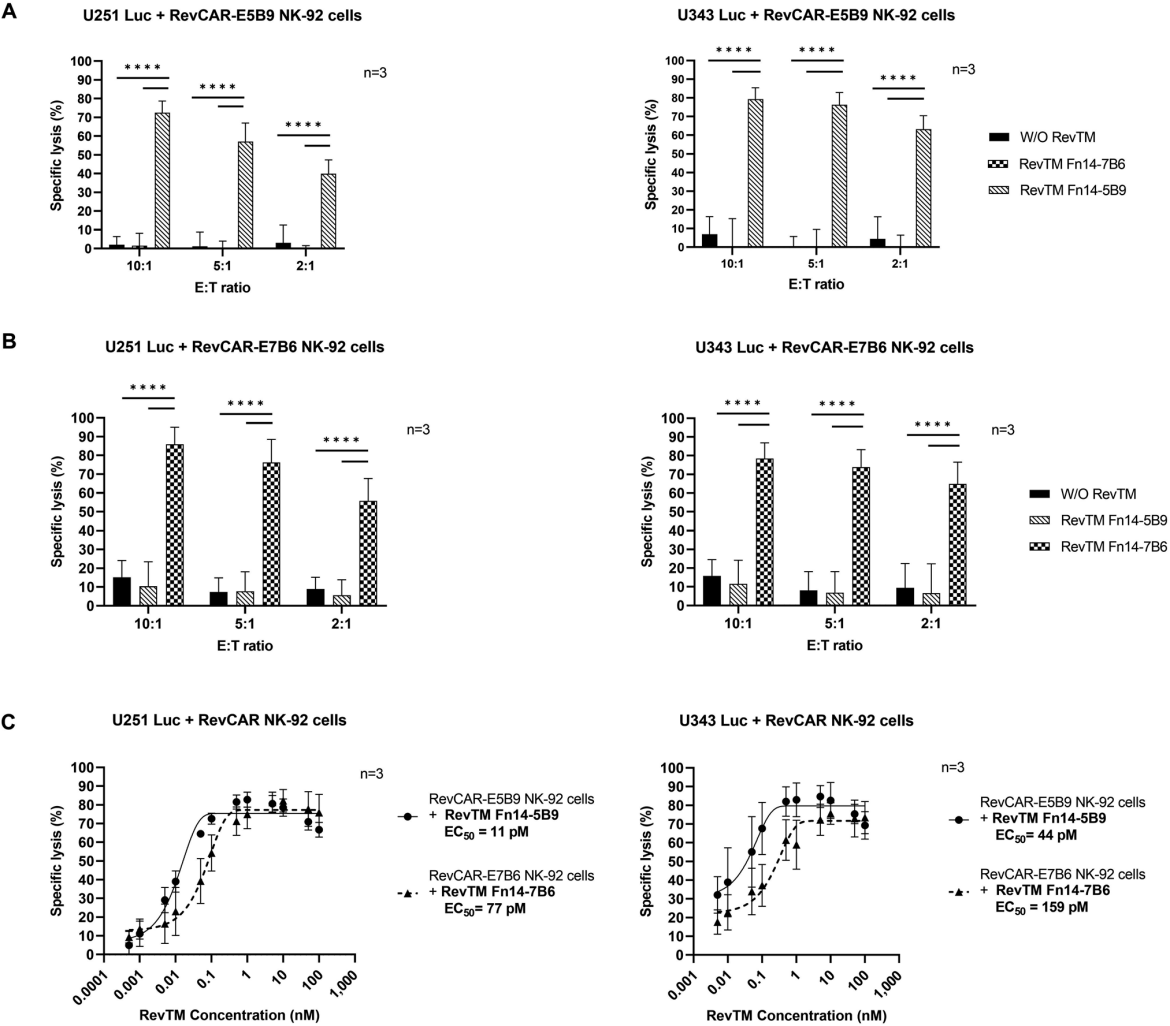
Specific lysis of GBM cells by Fn14-redirected RevCAR NK-92 cells. U251 Luc and U343 Luc cells were co-cultured with either (**A**) RevCAR-E5B9 or (**B**) RevCAR-E7B6 NK-92 cells at E:T cell ratios of 10:1, 5:1 and 2:1 in the absence or presence of RevTMs (50 nM) for 4 h. Cytotoxic activity was measured using a luminescence-based cytotoxicity assay. **C** Half-maximal effective concentration (EC_50_) of the Fn14-specific RevTMs was estimated. In the presence of a concentration range of the tested RevTMs, RevCAR NK-92 cells were co-cultured with the target cells at an E:T ratio of 10:1 for 4 h. **A**–**C** Data are shown for three independent experiments and represented as mean ± SD (one-way ANOVA with Tukey multiple comparisons test, ****p < 0.0001; comparison to samples without RevTM or with irrelevant RevTM).

Besides RevCAR T cells, we validated our RevCAR technology with NK-92 cells as a potential “off-the-shelf” living drug for targeting of GBM cells. RevCAR NK-92 cells were combined with the same Fn14-directed RevTMs. As depicted in Fig. 5A, B, RevCAR NK-92 cells were able to lyse both U251 Luc (A) or U343 Luc (B) cells at different E:T ratios (10:1, 5:1 and 2:1) only in the presence of the matching Fn14-specific RevTM, whereas a negligible lysis background was observed in conditions lacking the RevTM or having the non-matching one. The killing efficacy depends on the E:T ratio, whereby a significant lysis was even detected at the lowest E:T ratio of 2:1.

Furthermore, we determined the EC_50_ values for the RevTMs in the RevCAR NK-92 system. Therefore, RevCAR-E5B9 or RevCAR-E7B6 NK-92 cells were co-cultured with either the GBM cells and in the presence of the respective RevTM Fn14-5B9 or RevTM Fn14-7B6 at a gradient of concentrations. Both RevTMs effectively worked in the pM range (Fig. 5C). With both U251 Luc and U343 Luc GBM cells, the RevTM Fn14-5B9 had a 7- and 3.6- fold lower EC_50_ value, respectively, than the RevTM Fn14-7B6.

**Fig. 6:**
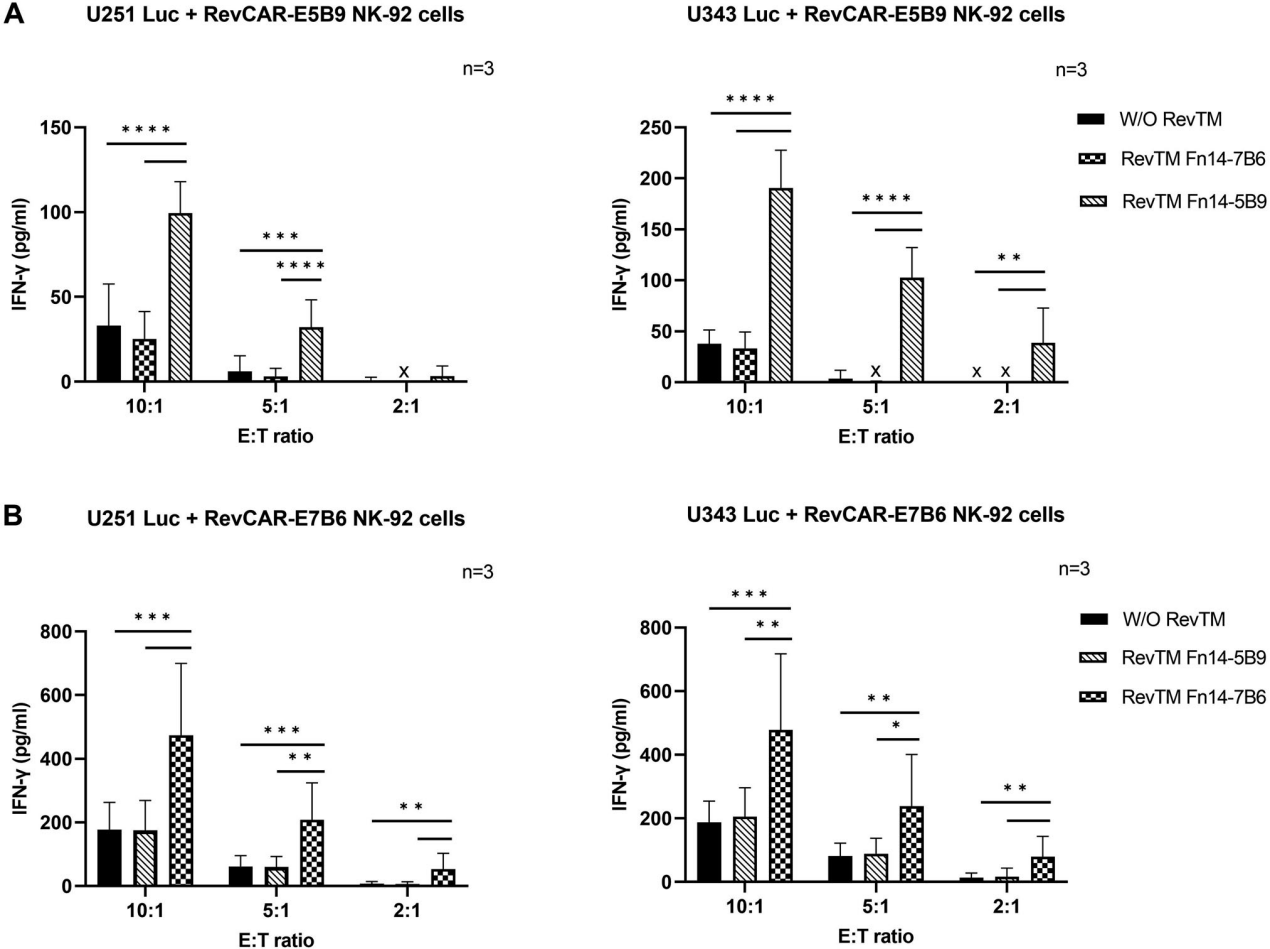
Cytokine secretion of Fn14-redirected RevCAR NK-92 cells. The pro-inflammatory cytokine IFN-γ was detected using ELISA in supernatants from co-culture experiments of either U251 Luc or U343 Luc cells with either (**A**) RevCAR-E5B9 or (**B**) RevCAR-E7B6 NK-92 cells incubated at an E:T ratio of 10:1, 5:1 or 2:1 in the absence or presence of either matching Fn14-specific RevTM or irrelevant one (negative control) for 4 h. Results are shown as mean ± SD for three independent experiments. (One-way ANOVA with Tukey multiple comparison test, *p < 0.0332, **p < 0.0021, ***p < 0.0002, ****p < 0.0001; comparison to samples without RevTM or with irrelevant RevTM).

Similar to RevCAR T cells, we analyzed the cytokine profile of the activated RevCAR NK-92 cells. Since IFN-γ is one of the major secreted cytokines from activated NK cells [41, 42], we estimated its release by RevTM- redirected RevCAR NK-92 cells. As illustrated in Fig. 6, IFN-γ was significantly secreted by RevCAR NK-92 cells in the presence of the respective RevTM, whereas secretion was negligible in conditions without RevTM or with non-matching RevTM. The highest cytokine release was observed for the highest E:T ratio. In comparison to RevCAR-E5B9 (Fig. 6A), RevCAR-E7B6 (Fig. 6B) induced relatively higher cytokine release.

In conclusion, these findings showed that both RevCAR-E5B9 and RevCAR-E7B6 NK-92 cell systems work efficiently against GBM cells with respect to tumor cell killing and IFN-γ release in a RevTM-dependent manner.

### In vivo killing of GBM cells with Fn14-directed RevCAR NK-92 cells

**Fig. 7:**
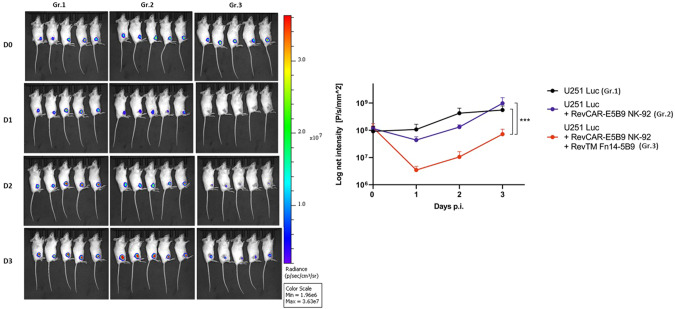
In vivo killing of GBM cells by Fn14-directed RevCAR NK-92 cells. Three groups of NXG mice were injected with either U251 Luc cells alone, U251 Luc cells and RevCAR NK-92 cells, or U251 Luc cells and RevCAR NK-92 cells in the presence of Fn14-specific RevTM. Luminescence was measured over 4 days after intraperitoneal application of luciferin. Quantitative luminescence intensity data are presented as mean values ± SD of five animals. Two-way ANOVA with Tukey’s multiple comparisons test was performed. (***p ≤ 0.001; comparison to mice groups with U251 Luc tumor cells alone or with RevCAR NK-92 cells but without RevTM). p.i post injection.

Following the in vitro GBM targeting with Fn14-redirected RevCAR NK-92 cells, a study with a heterotopic immunodeficient mouse model was conducted for validation of the immunotherapeutic effect of the system in vivo. Herein, three groups of mice were injected with U251 Luc cells either alone (group 1), with RevCAR-E5B9 NK-92 cells (group 2) or with RevCAR-E5B9 NK-92 cells plus RevTM Fn14-5B9 (group 3). Due to ethical reasons, we have limited the experiment to NK-92 cells, one tumor cell line and one RevCAR construct to show for the first time a proof-of-functionality of the RevCAR NK-92 technology in vivo. As shown in Fig. 7, a significant drop in the tumor luminescence signal was observed in mice of group 3, in comparison to the control groups, starting already at day 1 post injection (p.i.). These results validate the killing of GBM cells by the Fn14-redirected RevCAR NK-92 system in vivo.

## Discussion

Different ongoing clinical trials are currently testing conventional CAR T cells and recently also NK-92 cells to target different TAAs in GBM [43–45]. For example, clinical trials targeting the epidermal growth factor receptor variant III (EGFRvIII) showed promising outcome [46–48] but replicable responses were challenging to be obtained [47]. Additionally, the median progression-free survival was only 1 month [48] and adverse events, primarily associated with lymphodepleting chemotherapy were reported, including severe dyspnea and neurological changes [46]. Another trial targeting interleukin-13 receptor α chain variant 2 (IL13Rα2) showed fewer adverse events, but overall responses were limited [49]. A trial targeting HER2 in GBM demonstrated safety and potential benefits. Additionally, preclinical investigations on Chlorotoxin (CLTX), derived from scorpion venom, showed promising results in a murine GBM model [50]. CLTX-based CAR T therapy is currently in an actively recruiting phase I clinical trial [51]. Although CAR T cell therapies are showing promising therapeutic effects, adverse effects, such as cytokine release syndrome (CRS), have been reported in most cases [47]. Interestingly, no dose-related toxicity was observed in the first ongoing phase I clinical trial CAR2BRAIN (NCT03383978) using intrathecally administered HER2-specific-CAR-NK-92 cells against recurrent HER2^+^GBM [52]. Moreover, CAR NK-92 cells can be easily expanded for relatively long periods in culture, and could be used as “off-the-shelf” therapy allowing a large-scale production [53].They can also kill tumor cells via both CAR-dependent and independent mechanisms with relatively low toxicity due to their short half-life in patients [48, 50]. On the downside, however, multiple infusions of CAR NK-92 cells are required [53, 54], whereas CAR T cells can persist up to several years in patients [55]. Taken together, both CAR T and CAR NK-92 cell-based therapies have their respective advantages and disadvantages.

While GBM often shares common clinical features and histological characteristics, it has been proven to be genetically diverse requiring individual treatment regimens [56]. Due to the heterogeneity of GBM, immunotargeting of GBM might need the targeting of more than one TAA either sequentially or in parallel (multiple targeting also known as OR-gated targeting). Especially for the purpose of personalized medicine, we developed the versatile and switchable RevCAR system [15, 16, 28]. In these studies, we have shown how the universality of the RevCAR system allows adding or exchanging of anti-GBM RevTMs with different target specificities, structures, sizes, affinities, and pharmacokinetic properties. Using the same RevCAR effector cells, we were already able to target the TAAs GD2 and EGFR which can be overexpressed in GBM [29]. Here, we show for the first time that we can additionally target Fn14 on the same GBM cells using the RevCAR system. Perspectively, for targeting of GBM cells, Fn14 might be included in an OR-gated logic targeting strategy using the RevCAR system [29]. This flexibility of the RevCAR system provides a great advantage in targeting such a highly heterogenic tumor as GBM because it is not only cost- and time-effective, but it also offers the chance to overcome tumor escape, to achieve long-term anti-tumor effectiveness and to increase tumor specificity. As an additional advantage, the safety-switch of the RevCAR system can be used to stop adverse side effects and to minimize on-target/off-tumor toxicities in patients, unlike other CAR-based strategies [12, 23].

In the current study, we aimed to target the attractive TAA Fn14, because it is known to be overexpressed on GBM [34, 35]. Since a significant relation between high Fn14 expression and poor tumor prognosis has been previously reported in gliomas [35] and with a recent study supporting the therapeutic potential of Fn14-specific bispecific Ab and CAR-T/IL-15 cells against GBM [40], Fn14 appears as a potential target for CAR-based immunotherapies. However, like all other potential GBM targets for immunotherapy present on brain tumors, Fn14 is also expressed by normal brain cells [34, 36]. Although the expression level of Fn14 is relatively low on healthy brain tissue, the application of conventional CAR T cells is limited as even a low level of expression can lead to on-target/off-tumor toxicity. Such side effects, however, can be managed by using switchable adapter CARs such as the RevCAR platform in which the reactivity of CAR modified immune effector cells can be controlled by adjusting or stopping the administration of rapidly eliminated TMs [19, 29, 57, 58].

In a first step, we wanted to confirm that RevCAR T cells can be redirected against Fn14-expressing GBM cells. Next, we wanted to investigate whether RevCARs can be also functionally expressed in NK-92 cells in principal and, in particular, for targeting of Fn14-positive GBM cells. For that purpose, we designed and characterized two RevTMs against Fn14.

As mentioned already above, unlike other adapter CAR systems, RevCARs have an artificial epitope (E5B9 or E7B6) instead of an extracellular Ab domain [15], which circumvents unspecific binding and especially tonic signaling effects caused by complex formation of neighboring scFvs [15, 27]. Bearing in mind that (I) RevTMs have the same structure as (clinically used) bispecific Abs (Bispecific T cell Engager), and (II) the pharmacology of such bispecific Abs does not dramatically differ from the scFv-based UniCAR TMs (although the molecular weight of UniCAR TMs is slightly lower compared to bispecific Abs), the here described bispecific RevTMs are also expected to have pharmacokinetics similar to the scFv-based TMs of our previously described UniCAR platform, for which a proof of functionality and switchability was shown recently in an ongoing clinical trial (phase 1a trial-NCT04230265) [12, 13, 21, 29]. Moreover, we have shown in this study as well as in previous studies, that it is possible to steer the activity of RevCAR effector cells based on the availability and concentration of RevTMs [15, 16, 29]. In the current study, both RevCAR-E5B9 and RevCAR-E7B6 were able to induce high target-specific lysis of Fn14-expressing GBM cells associated with cytokine secretion in a RevTM- and target-dependent manner. Based on our killing measurements using target cell lines expressing different Fn14 levels, we conclude that the RevCAR system response is TAA dependent and requires a certain TAA threshold which might be beneficial for their discrimination between TAA^low^-expressing normal cells and TAA^high^-expressing tumor cells and, consequently, might reduce off-target toxicities. Interestingly, both modified T and NK-92 cells showed comparable efficiency in killing. Furthermore, we validated the observed in vitro efficient elimination of Fn14-positive GBM cells by steered RevCAR-NK-92 cells in vivo. In agreement with other studies [59–64], we have used here a heterotopic mouse model to provide an initial proof-of-concept for the immunotherapeutic functionality of our Fn14-specific RevCAR approach. As a next step in prospective studies, we aim to confirm the immunotherapeutic potential of the RevCAR system in a more clinically relevant GBM animal model mimicking the GBM tumor microenvironment and considering the crossing route of the blood-brain barrier (BBB), as well as, to further investigate long-term T cell differentiation in such setting.

In summary, the here presented data show for the first time that not only T cells, but also NK-92 cells can be modified with RevCARs and that the resulting RevCAR-expressing immune cells can be used for targeting of Fn14-positive GBM cells using the novel anti-Fn14 RevTMs with high target-specificity and tumor killing efficiency. Thus, both RevCAR T and RevCAR NK-92 systems are attractive and promising candidates for the treatment of GBM patients.

## Supplementary information

Figure Legend Supplemental Fig. 1

Supp. Fig. 1

## Data Availability

All relevant data are available from the authors upon request.
