# Supplementary material for: RevCAR-expressing immune effector cells for targeting of Fn14-positive glioblastoma
Source: Cancer Gene Ther. 2024 Apr 6;31(9):1323–34. doi: 10.1038/s41417-024-00766-8 (PMC11405279; doi:10.1038/s41417-024-00766-8)
Supplement: Supplementary file 1 — Figure Legend Supplemental Fig. 1 [file 41417_2024_766_MOESM1_ESM.docx]

**Supplementary Fig.1: Specific lysis of high-, low- and negative-Fn14-expressing target cells by Fn14-redirected RevCAR T cells.** **(A)** The expression level of Fn14 on luciferase (Luc)-expressing HEK293T Luc and Nalm6 Luc cells was determined by staining with anti-Fn14 mAb (primary Ab) and AlexaFluor647-conjugated anti-mouse IgG (secondary Ab). Flow cytometry data are displayed in histograms (light lines: negative control, dark lines: stained cells). The number of Fn14 antigens expressed per cell was detected by a bead-based flow cytometry assay (QIFIKIT). Quantitative data from two different experiments are shown as mean ± SD. **(B)** The specific lysis of high Fn14-expressing U251 Luc, Fn14 low-expressing HEK293T Luc and Fn14 negative Nalm6 Luc cells by Fn14-redirected RevCAR T cells was determined. Target cells were co-cultured individually with RevCAR-E5B9 T cells at E:T ratio of 1:2 or 5:1 in the absence or presence of RevTM Fn14 (50 nM) for 20 h. Cytotoxic activity was measured using a luminescence-based cytotoxicity assay. Data are shown for two independent T cell donors and represented as mean ± SD.
