# Supplementary figures and images for: RevCAR-expressing immune effector cells for targeting of Fn14-positive glioblastoma
Source: Cancer Gene Ther. 2024 Apr 6;31(9):1323–34. doi: 10.1038/s41417-024-00766-8 (PMC11405279; doi:10.1038/s41417-024-00766-8)

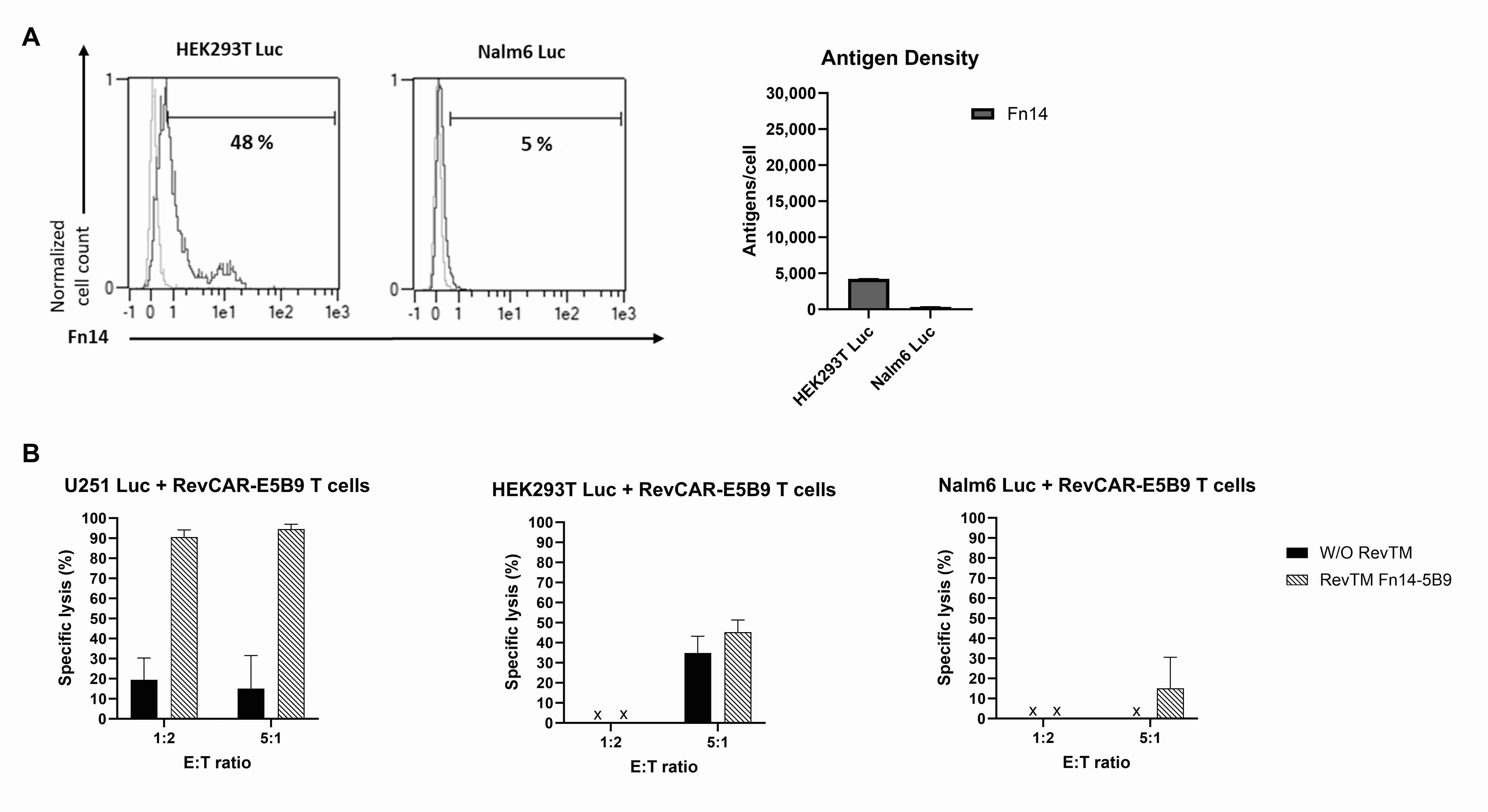

Supplement: Supplementary file 2 — Supp. Fig. 1 [file 41417_2024_766_MOESM2_ESM.jpg]
